# Advances in Composite Bioactive Scaffolds for Alveolar Bone Repair: Implications for Oral Surgery

**DOI:** 10.1590/0103-644020256728

**Published:** 2026-01-19

**Authors:** Wilman Rante Marampa, Nina Djustiana, Renny Febrida

**Affiliations:** 1 Master of Dental Science, Faculty of Dentistry, Universitas Padjadjaran, Jl. Sekeloa Sel. I No. 1, Lebakgede, Coblong District, Bandung City, 40132, West Java, Indonesia; 2 Department of Dental Materials Science and Technology, Faculty of Dentistry, Universitas Padjadjaran, Jalan Raya Bandung-Sumedang KM 21, Jatinangor, Sumedang 45363, Indonesia; 3 Oral Biomaterial Study Center, Universitas Padjadjaran, Jl. Sekeloa Sel. I No. 1, Lebakgede, Coblong District, Bandung City, 40132, West Java, Indonesia

**Keywords:** composite scaffolds, bioactive materials, alveolar bone regeneration, mandibular bone repair, bone tissue engineering

## Abstract

Craniofacial bone defects present a unique challenge due to the complex anatomical and functional demands of the region. Composite bioactive scaffolds have emerged as promising strategies for bone regeneration, combining the structural support of inorganic materials with the biological responsiveness of natural or synthetic polymers. This review discusses recent advancements in composite scaffolds tailored for alveolar and maxillofacial bone repair, with an emphasis on material selection, scaffold architecture, bioactivity, and therapeutic delivery capabilities. Key features, including porosity, mechanical strength, degradation rate, and anatomical adaptability, are evaluated for their clinical relevance. Furthermore, the roles of ion release, surface topography, and incorporation of growth factors or stem cells in modulating osteogenesis, angiogenesis, and immune responses are highlighted. Recent innovations also include dual-functional scaffolds that can promote bone formation while delivering antimicrobial or anti-inflammatory agents. Although most remain in preclinical stages, some composite scaffolds, particularly those based on hydroxyapatite, β-tricalcium phosphate (β-TCP), and bioactive glass, have progressed to clinical applications in dental implantology. The integration of biomimetic cues, 3D printing, and controlled drug release marks a significant step forward in personalized bone tissue engineering.

## Resumo

Defeitos ósseos craniofaciais representam desafio único devido às complexas exigências anatômicas e funcionais da região. *Scaffolds* bioativos compósitos surgem como estratégias promissoras para a regeneração óssea, combinando o suporte estrutural de materiais inorgânicos com a responsividade biológica de polímeros naturais ou sintéticos. Esta revisão discute avanços recentes em *scaffolds* compósitos desenvolvidos para a reparação óssea alveolar e maxilofacial, com ênfase na seleção de materiais, na arquitetura do *scaffold*, na bioatividade e na capacidade de entrega terapêutica. Características-chave, incluindo porosidade, resistência mecânica, taxa de degradação e adaptabilidade anatômica, são avaliadas por sua relevância clínica. Além disso, destacam-se os papéis da liberação de íons, da topografia de superfície e da incorporação de fatores de crescimento ou de células-tronco na modulação da osteogênese, da angiogênese e das respostas imunes. Inovações recentes incluem também *scaffolds* de dupla função, capazes de promover a formação óssea enquanto liberam agentes antimicrobianos ou anti-inflamatórios. Embora a maioria ainda se encontre em fases pré-clínicas, alguns *scaffolds* compósitos, particularmente aqueles baseados em hidroxiapatita, β-fosfato tricálcico (β-TCP) e vidro bioativo, já avançaram para aplicações clínicas na implantodontia. A integração de sinais biomiméticos, a impressão 3D e a liberação controlada de fármacos representam um avanço significativo na engenharia de tecidos ósseos personalizada.

**Figure:**
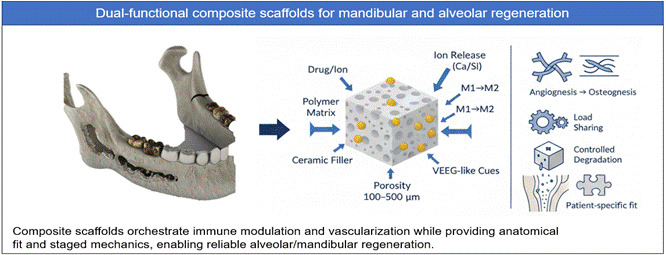

## Introduction

The oral and maxillofacial bones, including the mandible, maxilla, and alveolar processes, possess unique anatomical and physiological characteristics distinct from those of systemic skeletal bones. These bones are not only responsible for supporting mastication, speech, and facial aesthetics, but they also exist in a highly dynamic environment that is exposed to continuous mechanical stress, microbial colonisation, and inflammation. Bone regeneration in this region involves tightly regulated phases, namely inflammation, repair, and remodelling, driven by interactions among osteogenic, immune, and vascular cells. Therefore, materials intended for bone repair in the oral-maxillofacial region must be tailored to meet these region-specific demands. Reconstruction of alveolar and mandibular bone defects remains one of the most challenging issues in oral and maxillofacial surgery (OMFS), commonly arising from trauma, tumour resection, congenital anomalies, or chronic infections. These defects significantly impair oral function and aesthetics, reducing patients’ quality of life and often causing a substantial psychological burden ^1^. Epidemiological data indicate that maxillofacial trauma affects a significant global population, with surgical treatment rates reaching up to 25.1 per 100,000 individuals annually in countries such as Germany ^2^. Mandibular fractures alone account for nearly 47% of maxillofacial injuries, particularly in males aged 20-40 years in developing regions such as India ^3^. In specific trauma centres, maxillofacial injuries make up 18.5% of all trauma-related hospital admissions ^4^.

Traditional approaches such as autografts and allografts have long served as the clinical gold standard for bone reconstruction due to their osteoconductive and osteoinductive properties. However, they are constrained by factors like limited graft availability, donor site morbidity, immune rejection, and unpredictable resorption rates ^5^ ^,^ ^6^. More importantly, these grafts often fall short in addressing the aesthetic, structural, and biological complexity of maxillofacial reconstruction, which requires materials that can conform to irregular bone geometries and integrate seamlessly with surrounding soft tissues ^6^. To overcome these limitations, the field of tissue engineering has introduced bioactive composite scaffolds, engineered materials designed to mimic the extracellular matrix while supporting cellular adhesion, proliferation, and differentiation. These scaffolds commonly consist of organic polymers, such as chitosan, gelatin methacryloyl (GelMA), and polycaprolactone (PCL), combined with inorganic fillers, including hydroxyapatite (HA), β-tricalcium phosphate (β-TCP), or bioactive glass ^7^ ^,^ ^8^ ^,^ ^9^ ^,^ ^10^. The synergy between these components facilitates not only osteoconduction and mechanical stability but also drug delivery and immune modulation ^11^ ^,^ ^12^. Recent innovations have brought forth promising scaffold designs for mandibular and alveolar bone regeneration. For instance, Yao et al. (2024) developed a collagen/gelatin/nano-β-TCP scaffold with high porosity (84-94%), which exhibited superior biodegradability and osteogenic differentiation, making it suitable for alveolar repair ^13^. Kim et al. (2024) introduced a customised 3D-printed polydiolcitrate-hydroxyapatite scaffold infused with graphene oxide hydrogel that enhanced extracellular matrix mineralisation and angiogenesis in vivo ^14^. He et al. (2025) proposed an injectable hydrogel combining puerarin, chitosan, and mesoporous silica nanoparticles, offering dual functionality in bone regeneration and inflammation control ^15^. Despite the promising advances, current reviews rarely focus on the specific challenges and considerations associated with oral-maxillofacial bone defects ^16^ ^,^ ^17^. Most literature generalises findings from orthopaedic or craniofacial applications, often neglecting OMFS-specific parameters such as aesthetic restoration, functional loading, and integration with soft tissues ^18^ ^,^ ^19^ ^,^ ^20^. Therefore, this review aims to ^1^ summarise the current development of bioactive composite scaffolds for mandibular and alveolar bone regeneration; ^2^ highlight limitations in scaffold design, performance, and translational application; and ^3^ explore the future of dual-functional scaffolds with regenerative and therapeutic capabilities tailored explicitly to the oral-maxillofacial region. A thorough understanding of the biological processes, mechanical demands, and design requirements in this region is crucial for developing next-generation scaffolds that can facilitate both functional and aesthetic bone reconstruction.

### Scientific basis of composite bioactive scaffolds

The development of composite bioactive scaffolds for alveolar and mandibular bone regeneration is grounded in a combination of material science, biological principles of osteogenesis, and the unique anatomical and biomechanical requirements of the maxillofacial region ^21^ ^,^ ^22^ ^,^ ^23^. Effective scaffold design must replicate both the structural and functional properties of native bone to support cellular attachment, proliferation, differentiation, vascularisation, and eventual tissue integration ^24^ ^,^ ^25^ ^,^ ^26^.

### Key Biological Principles in Bone Regeneration

Successful bone regeneration is governed by a finely coordinated interplay of biological processes: osteoconduction, osteoinduction, osteointegration, and angiogenesis ^27^ ^,^ ^28^ ^,^ ^29^. These mechanisms are especially relevant in oral and maxillofacial regions, where intramembranous ossification dominates, contrasting with endochondral ossification seen in long bones. This distinction influences scaffold selection, as the former demands early vascularization and direct osteoblast recruitment for successful regeneration ^30^ ^,^ ^31^ ^,^ ^32^.

Modern bioactive composite scaffolds aim to replicate this complex environment through tailored material design. The ideal scaffold incorporates osteoconduction, where materials such as β-TCP or hydroxyapatite provide a passive framework guiding new bone growth along the scaffold structure ^33^ ^,^ ^34^. Simultaneously, osteoinduction is achieved via delivery of bioactive molecules or ions such as BMPs, Ca²⁺, and Si⁴⁺ to stimulate differentiation of progenitor cells into osteoblasts ^35^ ^,^ ^36^. Furthermore, angiogenesis is facilitated by factors like VEGF or by incorporating calcium phosphate derivatives known to promote endothelial cell migration and neovascularization ^37^ ^,^ ^38^ ^,^ ^39^. Composite scaffolds further enhance this process by combining organic polymers such as collagen and gelatin with inorganic bioceramics, including nano-β-TCP and HA. These materials mimic the bone extracellular matrix and offer spatial and biochemical cues for cell attachment, nutrient exchange, and mechanical support. Beyond material selection, scaffold architecture, including porosity, pore size, and interconnectivity, plays a critical role. Scaffolds with pore sizes between 100-500 µm have been shown to support optimal vascular ingrowth and osteoid deposition in mandibular models. Recent innovations include dual-functional scaffolds that simultaneously support osteogenesis and deliver therapeutic agents, such as anti-inflammatory drugs or antibiotics, enhancing both regeneration and infection control as demonstrated in clinical and experimental studies. ^40^ ^,^ ^41^ ^,^ ^42^ ^,^ ^43^ ^,^ ^44^ ^,^ ^45^

Beyond osteoconduction/osteoinduction, early immune cues strongly condition alveolar healing. Transient M1-like macrophage activity recruits and instructs progenitors, while timely polarization toward M2 phenotypes supports resolution, angiogenesis, and matrix deposition. Composite scaffolds can bias this axis via surface charge/topography, Ca/Si ion dissolution (e.g., from BG/β-TCP), and local delivery of immunoregulatory small molecules-collectively dampening excessive IL-6/TNF-α while elevating pro-healing mediators (e.g., IL-10, OPG). Designing scaffolds as *immuno-instructive* rather than passive carriers aligns with recent co-culture and in vivo data showing that macrophage-MSC crosstalk is decisive for osteogenesis in mineralized matrices. ^46^ ^,^ ^47^

In intramembranous repair, angiogenesis precedes and sustains osteogenesis; early microvascular ingress (favored by 100-500 µm interconnected pores and VEGF-mimetic cues) supplies oxygen and perivascular niches that stabilize RUNX2-positive lineages. ^48^ ^,^ ^49^ Functionalization and pore architecture should therefore be staged to secure perfusion, then sustain osteoblast maturation, while synchronizing degradation to preserve load transfer as bone consolidates. ^50^ ^,^ ^51^

**Figure 1:**
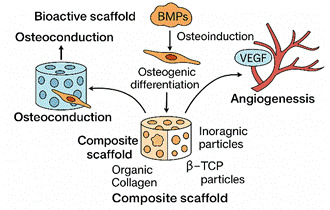
Schematic Representation of Key Biological Processes in Alveolar Bone Regeneration A conceptual diagram illustrating the roles of osteoconduction, osteoinduction, and angiogenesis in bone regeneration. Bioactive scaffolds incorporating components such as β-TCP, BMPs, VEGF, and collagen are designed to guide bone growth, stimulate osteogenic differentiation, and promote neovascularization. Composite scaffolds integrate these functions to enhance alveolar bone repair.

**Figure 2:**
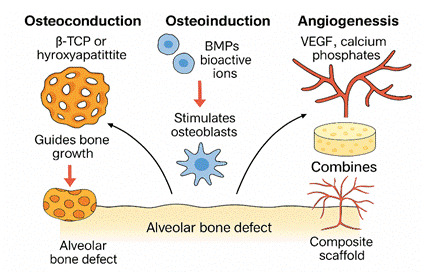
Mechanistic Contributions of Bioactive Scaffold Components in Alveolar Bone Defect Healing. An illustrative breakdown of how osteoconduction (via β-TCP or hydroxyapatite), osteoinduction (via BMPs and bioactive ions), and angiogenesis (via VEGF and calcium phosphates) contribute individually to alveolar bone regeneration. These mechanisms converge within the composite scaffold to create a conducive microenvironment for osteogenesis and neovascularization.

**Figure 3:**
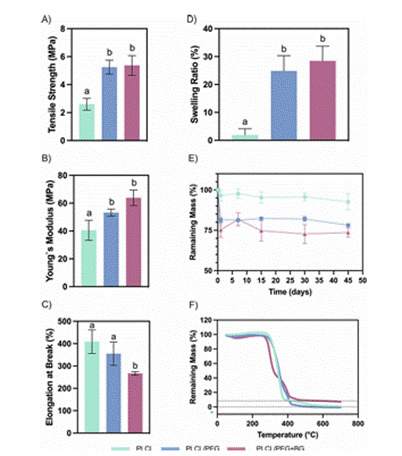
Mechanical and degradation properties of electrospun PLCL/PEG/BG composite scaffold. Data include tensile strength, Young’s modulus, swelling ratio, and degradation profile over time. - Reproduced from de Souza et al., 2024, *J Biomed Mater Res B*, under CC BY 4.0. ^62^

### Material Composition and Functional Integration in Composite Scaffolds

Composite scaffolds are increasingly engineered by combining organic polymer matrices such as chitosan and polycaprolactone with inorganic fillers, including hydroxyapatite or bioactive glass. This dual-phase architecture is designed to replicate the mechanical strength and hierarchical porosity of natural bone while providing a favorable microenvironment for cell adhesion and osteogenic differentiation ^7^ ^,^ ^8^ ^,^ ^12^ ^,^ ^52^. The organic polymeric phase encompasses both natural and synthetic polymers, each offering distinct advantages. Natural polymers such as gelatin and chitosan support cell adhesion and proliferation due to the presence of cell-interactive sequences, including RGD (Arg-Gly-Asp) motifs. These materials also provide intrinsic biocompatibility and facilitate nutrient diffusion ^53^ ^,^ ^54^. Conversely, synthetic polymers like polycaprolactone (PCL) and poly(lactic-co-glycolic acid) (PLGA) offer superior mechanical integrity and controlled biodegradability, making them excellent carriers for mineral phase incorporation and localized drug delivery ^55^ ^,^ ^56^. The inorganic mineral phase primarily consists of calcium phosphate-based ceramics such as hydroxyapatite (HA) and β-tricalcium phosphate (β-TCP) that closely mimic the mineral component of natural bone ^13^ ^,^ ^57^. HA is recognized for its high osteoconductivity and mechanical stability, whereas β-TCP exhibits a faster resorption rate, gradually releasing calcium and phosphate ions that stimulate osteoblast differentiation ^58^ ^,^ ^59^. Additionally, bioactive glass and calcium carbonate (CaCO₃) contribute further by promoting ion dissolution, enhancing angiogenesis, and facilitating extracellular matrix remodeling in bone regeneration sites ^60^ ^,^ ^61^. Recent innovations demonstrate the incorporation of chlorinated bioactive glass into PLCL/PEG electrospun scaffolds. De Souza et al. (2024) developed a composite scaffold containing 15% BG, which significantly enhanced tensile strength and hydrophilicity while supporting alveolar bone-derived MSC viability and mineralization in experimental studies ^62^. The reduction in fiber diameter (approximately 436 nm) and increased swelling ratio suggest improved cellular integration and biodegradation control, which are essential for maxillofacial scaffolds tailored to irregular geometries and remodeling rates.

The dispersion uniformity, interfacial bonding, and mass ratio between organic and inorganic phases are critical parameters that influence scaffold porosity, mechanical strength, and degradation behavior. For instance, increasing β-TCP content above 60% can improve osteogenesis but may reduce flexibility and printability during fabrication ^52^ ^,^ ^56^ ^,^ ^63^. The most promising composite strategies often leverage the synergy between different polymers to overcome the trilemma of mechanical strength, bioactivity, and a cell-friendly environment.

Polycaprolactone exemplifies this challenge, as it is valued for its good mechanical strength and slow degradation rate, making it an excellent candidate for long-term, load-bearing scaffolds (64, 65, 66). However, PCL is inherently bio-inert and hydrophobic, which limits cell attachment and infiltration, and its acidic degradation products can trigger an inflammatory response ^67^. In contrast, gelatin methacryloyl (GelMA) is highly biomimetic, containing RGD sequences that promote cell adhesion and MMP-cleavable sites, allowing cells to remodel their surrounding matrix. However, GelMA's poor mechanical strength makes it unsuitable for load-bearing applications independently ^68^ ^,^ ^69^. By combining PCL, GelMA, and Bioactive Glass (BG) in an advanced hybrid system, these limitations can be effectively overcome. In designs such as core-shell nanofibers with PCL core and GelMA shell embedded within a GelMA/BG hydrogel, the PCL provides a robust mechanical framework, the GelMA shell creates a cell-friendly and well-integrated interface, and the dispersed BG particles provide the necessary ionic cues for osteogenesis and angiogenesis. This hierarchical system is simultaneously strong, bioactive, and highly supportive of cellular regeneration, as demonstrated by an improved compressive modulus (approximately 353 kPa) and better stability compared to the hydrogel alone ^70^.

### Positional Memory and Embryological Considerations in Scaffold Application

Bone regeneration in the maxillofacial region demands a nuanced understanding of the embryological origin of skeletal tissues. Unlike long bones derived from the lateral plate mesoderm, both the maxilla and mandible originate from the cranial neural crest. This distinction has profound functional consequences for regenerative approaches. Leucht et al. (2008) described a phenomenon known as "positional memory," wherein neural crest-derived skeletal progenitor cells demonstrated preferential healing in craniofacial defects, while mesoderm-derived cells failed to differentiate appropriately when grafted into mandibular sites, instead forming cartilage rather than bone ^71^ ^,^ ^72^. This finding highlights the critical importance of selecting region-specific progenitor cells or developing scaffold microenvironments that accurately replicate the native bone niche characteristics. Furthermore, the embryological origin influences not only cellular behavior but also the regenerative capacity and healing patterns observed in maxillofacial reconstruction. Wu et al. (2019) emphasized that successful bone regeneration depends not only on osteogenesis but also on concurrent neovascularization, representing a dual requirement that should be addressed simultaneously in scaffold design ^73^. This integrated approach becomes particularly crucial in the maxillofacial region, where the complex anatomical architecture and functional demands necessitate scaffolds that can support both rapid vascularization and appropriate osteogenic differentiation while respecting the inherent positional memory of neural crest-derived tissues.

Craniofacial progenitors retain region-specific transcriptional/epigenetic programs (e.g., neural-crest Hox status and chromatin accessibility) that shape lineage choice and matrix deposition. ^74^ ^,^ ^75^ ^,^ ^76^ Practically, scaffolds for mandibular/maxillary sites should be tuned for these cues, which present neural-crest-relevant ECM motifs, stiffness ranges, and Wnt/Notch-compatible microenvironments to recapitulate the native niche. ^77^ ^,^ ^78^ ^,^ ^79^ ^,^ ^80^ Craniofacial vasculature exhibits distinctive perivascular support (notably neural-crest-derived pericytes) and dense plexus architecture under cyclical oral loading. Vascularization strategies that recruit both endothelial and perivascular cells (e.g., SDF-1α gradients, ion-mediated pro-angiogenic signaling) are therefore desirable in maxillofacial scaffolds. ^81^ ^,^ ^82^ ^,^ ^83^

### Scaffold Properties Required for Maxillofacial Bone Repair

In the context of OMFS, scaffold design must satisfy stricter biological and mechanical demands compared to general orthopedic applications. This includes adaptation to the dynamic environment of the jaw, frequent exposure to masticatory forces, and the need for comprehensive soft tissue integration. The unique anatomical and functional requirements of the maxillofacial region necessitate scaffolds that can withstand complex loading patterns while maintaining biocompatibility and promoting regeneration in areas with limited vascular supply.

**Box 1:**
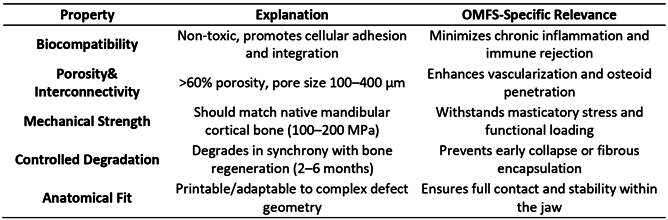
Critical Scaffold Properties for Maxillofacial Bone Repair ^(84^ ^,^ ^85^ ^,^ ^86^ ^,^ ^87^ ^,^ ^88^ ^,^ ^89^ ^,^ ^90^ ^,^ ^91^ ^,^ ^93^ ^,^ ^93^

Recent efforts to fabricate bioactive scaffolds with hierarchical porosity have emphasized the synergistic roles of macro-, micro-, and mesopores in promoting both osteogenesis and vascularization. This multi-scale pore architecture addresses different aspects of bone regeneration, where macropores facilitate cell migration and vascular ingrowth, micropores enhance protein adsorption and cellular attachment, and mesopores provide optimal surface area for ion exchange and nutrient transport. Gómez-Cerezo et al. (2021) demonstrated that combining 3D printing with porogen leaching and mesoporous bioactive glass incorporation yielded scaffolds with approximately 80-90% total porosity and 30% intra-strut microporosity, resulting in early mineralization and enhanced osteoblast activity ^94^.

The implementation of hierarchical pore architecture (Figure 4 - 5) represents a significant advancement in scaffold design, as it addresses the complex interplay between mechanical stability and biological functionality. The morphological changes observed in scaffold struts following porogen leaching and the internal structural modifications visualized through micro-CT analysis validate the necessity of this multi-scale approach in next-generation scaffolds for maxillofacial reconstruction. These findings demonstrate that hierarchical porosity not only enhances the quantitative increase in total porosity but also optimizes the spatial distribution of pores, creating an environment conducive to both immediate mechanical support and long-term biological integration**.** ^95^

Extrusion-based printing is GMP-friendly and scalable but offers moderate resolution; DLP achieves finer features and gradient porosity, with attention to photoinitiator residues and post-cure cytocompatibility; melt electrowriting yields ordered microfibers for GBR membranes but currently has lower throughput; porogen leaching adds intra-strut microporosity yet demands tight process control to ensure batch-to-batch reproducibility and sterilization validation. Method choice should be justified against clinical geometry, throughput, and regulatory readiness. With these process constraints mapped, the following section classifies composite systems and links composition to property windows and biological outcomes. ^96^ ^,^ ^97^ ^,^ ^98^

### Cellular Response to Bioactive Composites

Composite scaffolds influence cellular behavior through multiple interconnected mechanisms that collectively determine the success of bone regeneration. Surface chemistry and topography play fundamental roles in enhancing cell adhesion and proliferation by providing optimal binding sites and physical cues that guide cellular attachment and spreading. Simultaneously, ion release kinetics from the scaffold materials stimulate the differentiation of mesenchymal stem cells into osteoblasts through the controlled delivery of calcium, phosphate, and other bioactive ions. Additionally, the degradation products generated during scaffold resorption significantly influence local pH levels, immune modulation, and vascular remodeling processes that are crucial for tissue integration ^27^. Functionalization with bioactive molecules such as BMPs, VEGF, and antibiotics, or incorporation of mesenchymal stromal cells (MSCs), further enhances the regenerative response by providing specific biological signals that accelerate healing and prevent complications. These advanced approaches represent a shift toward more sophisticated, biologically informed scaffold design that addresses multiple aspects of the regeneration process simultaneously.

**Box 2:**
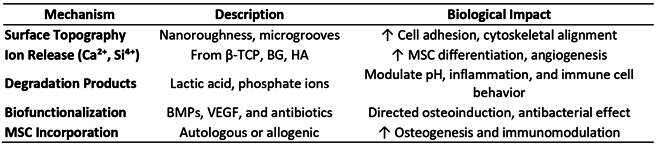
Cellular Mechanisms Influenced by Bioactive Composite Scaffolds ^26^ ^,^ ^36^ ^,^ ^38^ ^,^ ^39^ ^,^ ^44^ ^,^ ^45^ ^,^ ^84^ ^,^ ^85^ ^,^ ^89^

The immunomodulatory behavior of scaffolds is increasingly recognized as critical to successful bone regeneration, representing a paradigm shift in our understanding of scaffold function. Recent evidence by Kolliopoulos et al. (2025) demonstrated that macrophage-MSC interactions significantly influence osteogenesis within a mineralized collagen scaffold environment ^95^. Their innovative 3D co-culture platform revealed that MSCs licensed by M1 macrophages exhibited elevated expression of osteogenic markers, including RUNX2, ALPL, and OPN, particularly under indirect co-culture conditions. Furthermore, the inflammatory cytokines secreted by macrophages, such as IL-6 and OPG, were modulated by the scaffold environment, emphasizing the scaffold's active role in immunoregulation rather than serving as a passive structural support. These findings underscore the dual importance of scaffold composition and cellular interaction in modulating the inflammatory-osteogenic axis that is essential for maxillofacial bone healing. The recognition that scaffolds actively participate in immune regulation opens new avenues for designing materials that can orchestrate the complex interplay between inflammation and regeneration, ultimately leading to more predictable and successful clinical outcomes in oral and maxillofacial reconstruction.

### Classification and types of composite bioactive scaffolds

Composite scaffolds used in oral and maxillofacial surgery are broadly classified based on their constituent materials, notably the organic polymer matrix and inorganic bioactive filler. This section explores the major types of composite scaffolds, with an emphasis on their material composition and potential applications in alveolar and mandibular bone regeneration. Below, we link each composition to its dominant property window and expected biological outcome, and we indicate a practical translational readiness level (TRL) for maxillofacial use.

**Box 3:**
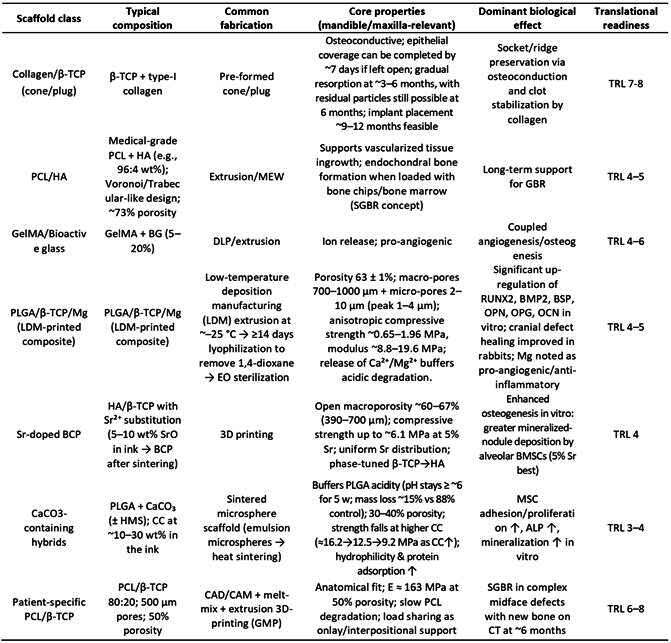
Translational readiness (TRL) of composite bioactive scaffolds for mandibular and alveolar bone regeneration ^99^ ^,^ ^100^ ^,^ ^101^ ^,^ ^102^ ^,^ ^103^ ^,^ ^104^ ^,^ ^136^

### Classification Based on Polymer Matrix

Composite scaffolds can be systematically categorized according to their polymer matrix composition, specifically whether they incorporate natural or synthetic polymers, each offering distinct advantages for maxillofacial bone regeneration applications. Natural polymers, including chitosan, collagen, gelatin, and alginate, are widely utilized due to their intrinsic biocompatibility, biodegradability, and inherent ability to mimic the extracellular matrix environment. These materials provide excellent biological recognition sites that facilitate cellular attachment and proliferation. For example, collagen is frequently combined with β-TCP or HA to enhance mechanical strength while retaining superior cell adhesion properties ^13^ ^,^ ^105^ ^,^ ^106^. These collagen-based combinations have demonstrated excellent osteoconductivity and hemostatic function, making them particularly well-suited for alveolar bone repair procedures where rapid healing and biological integration are paramount. Conversely, synthetic polymers such as PCL, PLGA, and PEG offer greater control over degradation kinetics and mechanical behavior, allowing for more predictable scaffold performance. The synthetic approach enables precise tuning of material properties to match specific clinical requirements. PEG-based scaffolds, when blended with HA or bioactive glass, have demonstrated excellent printability, dimensional stability, and enhanced suitability for load-bearing mandibular applications where mechanical demands are significantly higher ^107^ ^,^ ^108^ ^,^ ^109^. This synthetic approach proves particularly valuable in cases requiring complex geometries or extended mechanical support during the regeneration process.

The selection between natural and synthetic polymer matrices ultimately depends on the specific clinical scenario, with natural polymers favored for soft tissue integration and rapid biological response, while synthetic polymers are preferred for applications requiring precise mechanical control and extended structural support.

**Figure 6:**
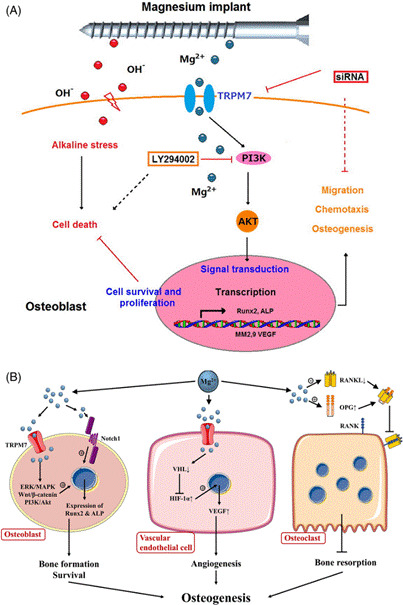
Proposed mechanisms of magnesium-induced osteogenesis. (A) Mg²⁺ ions released from magnesium implants enter osteoblasts via TRPM7 channels, triggering PI3K/AKT signaling, promoting cell survival and transcription of osteogenic markers such as Runx2 and ALP. (B) Mg²⁺ also regulates osteoblast differentiation, angiogenesis via VEGF upregulation in endothelial cells, and osteoclast inhibition through OPG/RANKL balance. Adapted with permission from Weng et al., 2023, Journal of Biomedical Materials Research Part B: Applied Biomaterials (DOI: 10.1002/jbm.b.35246).^121^

### Classification Based on Inorganic Bioactive Filler

The inorganic component of composite scaffolds plays a pivotal role in osteoinduction and structural integrity, with various bioactive fillers offering distinct advantages for maxillofacial bone regeneration. Common inorganic fillers include HA, β-TCP, CaCO₃, and bioactive glass, each contributing unique properties to the composite system ^110^ ^,^ ^111^. Hydroxyapatite represents the most extensively studied bioceramic due to its chemical similarity to natural bone mineral, enhancing osteoblast attachment and mineral deposition, although it demonstrates relatively slow resorption kinetics. In contrast, β-TCP degrades more rapidly and supports accelerated bone remodeling processes. Composite scaffolds containing β-TCP or HA combined with gelatin or chitosan have been widely implemented in alveolar ridge augmentation procedures ^112^ ^,^ ^113^ ^,^ ^114^. Recent developments in ion-doped biphasic calcium phosphate scaffolds further underscore the importance of compositional tuning for optimal biological response. Oliveira et al. (2024) fabricated Sr-enriched β-TCP/HA scaffolds via a straightforward 3D-printing method using SrCO₃, demonstrating that the addition of 5% SrO improved both mechanical strength and mineralized nodule deposition by aBMSCs ^115^. However, higher Sr content (10%) led to increased HA formation but reduced cell viability, emphasizing the critical need for optimal doping ratios in scaffold design to balance bioactivity with cytocompatibility.

Bioactive glass represents a particularly sophisticated class of inorganic fillers that release ions such as Ca²⁺ and Si⁴⁺, which critically stimulate osteogenic signaling pathways and promote angiogenesis ^86^. However, the specific formulation directly dictates its ion release profile and biological response. The three most studied formulations, including 45S5, S53P4, and 13-93, exhibit important differences in their clinical performance. 45S5, with its low silica and high sodium content, is highly reactive, leading to rapid ion release and a significant pH shift that can be cytotoxic at high concentrations ^116^. In contrast, S53P4 has a higher silica content, resulting in a more controlled dissolution rate and clinically proven antibacterial properties ^117^. The 13-93 formulation offers a middle ground, with rapid calcium release but a more moderate pH shift, demonstrating superior biological performance with lower cytotoxicity than 45S5 ^118^ ^,^ ^119^. At the molecular level, these released ions trigger specific intracellular signaling cascades that orchestrate bone regeneration. Calcium and magnesium ions activate two major signaling hubs: the Mitogen-Activated Protein Kinase/Extracellular signal-Regulated Kinase (MAPK/ERK) pathway and the Phosphoinositide 3-Kinase/Akt (PI3K/Akt) pathway, as demonstrated in recent molecular studies ^120^.

Activation of the MAPK/ERK pathway is crucial for osteoblast proliferation and differentiation, as well as new blood vessel formation ^121^. Meanwhile, the PI3K/Akt pathway, which is exceptionally responsive to magnesium ions, promotes cell survival and enhances the expression of key osteogenic transcription factors like RUNX2. The coordinated activation of these pathways represents the fundamental mechanism by which bioactive glass actively orchestrates tissue regeneration ^122^. Calcium carbonate is often considered a cost-effective filler with resorbable characteristics, suitable for non-load-bearing scaffolds. However, its behavior is highly dependent on its crystalline polymorphism, with the three primary forms, calcite, aragonite, and vaterite, exhibiting different stabilities and solubilities that directly impact their resorption rate and bioactivity. Calcite is the most stable and least soluble, while vaterite, the least stable, shows the highest bioactivity due to its ability to rapidly transform into hydroxyapatite in the presence of body fluids ^123^ ^,^ ^124^. Nevertheless, the primary limitation of CaCO₃ is its rapid and often unpredictable resorption rate, which can lead to graft failure and void formation if the material degrades faster than new bone formation ^125^. Therefore, the most promising role for CaCO₃ may be as a functional additive rather than a bulk structural material. Its alkaline nature can buffer the acidic byproducts of polymer degradation from materials like PCL, and its pH-sensitive solubility makes it an excellent carrier for targeted drug delivery, releasing therapeutic agents like antibiotics or growth factors in response to the acidic microenvironment of an injury site ^126^ ^,^ ^127^.

### Functional Hybrid Scaffolds and OMFS Applications

Recent research has highlighted multifunctional scaffolds that combine both regenerative and therapeutic roles, representing a paradigm shift toward more sophisticated clinical solutions. For instance, the collagen/gelatin/nano-β-TCP scaffold developed by Yao et al. (2024) was tailored explicitly for alveolar bone grafts due to its favorable microarchitecture and superior osteoconductivity ^13^. Similarly, Kim et al. (2024) created a 3D-printed polydiolcitrate-HA scaffold integrated with a graphene oxide hydrogel, optimized for calvarial and mandibular defects with enhanced vascularization capacity ^14^. He et al. (2025) engineered an injectable scaffold system using puerarin, chitosan, and mesoporous silica nanoparticles, creating a hydrogel scaffold that not only enhanced osteogenesis but also modulated inflammation, showing significant potential for clinical use in post-tumor defect reconstruction ^15^. Additionally, Ma et al. (2024) introduced a coaxial SIS/HA composite hydrogel scaffold functionalized with antibacterial GL13K and an exosome-loaded SIS hydrogel core, as demonstrated in recent experimental studies ^26^.

This innovative scaffold promoted alveolar bone regeneration after tooth extraction by enhancing both osteogenesis and angiogenesis while simultaneously reducing microbial load. This design exemplifies the future of dual-functional, patient-specific scaffold systems tailored to address the complex requirements of oral and maxillofacial surgery. Yu et al. (2024) introduced a particularly innovative injectable composite hydrogel scaffold composed of GelMA reinforced with bioactive glass (BG) and coaxial PCL@GelMA nanofibers ^70^. This multifunctional design was engineered to address both the structural and biological demands of maxillofacial bone regeneration through a sophisticated hierarchical architecture.

The incorporation of BG enhanced the scaffold's osteogenic and angiogenic capacity, while the coaxial nanofiber framework contributed to improved mechanical stability and sustained structural integrity within the defect site. Scanning electron microscopy analysis revealed that increasing BG content from 0% to 20% progressively modified the scaffold's surface architecture, producing more granulated and microstructured textures that were highly favorable for cell attachment and proliferation. The presence of aligned nanofibers further supported interconnectivity within the matrix, creating optimal pathways for nutrient transport and cellular migration. When tested in a rat critical-sized cranial defect model, this advanced scaffold promoted substantial new bone formation, achieving up to 25% regeneration within 12 weeks, along with significant upregulation of key osteogenic and angiogenic markers such as ALP, RUNX2, and CD31. ^70^ These findings underscore the potential of this composite scaffold as a truly dual-functional platform, capable of providing both mechanical reinforcement and biological stimulation for complex bone defects in the maxillofacial region. These examples collectively illustrate how composite scaffold design is rapidly evolving toward patient-specific, dual-functional platforms that comprehensively address the multifaceted requirements of maxillofacial bone regeneration, marking a significant advancement in the field of oral and maxillofacial surgery.

**Figure 7:**
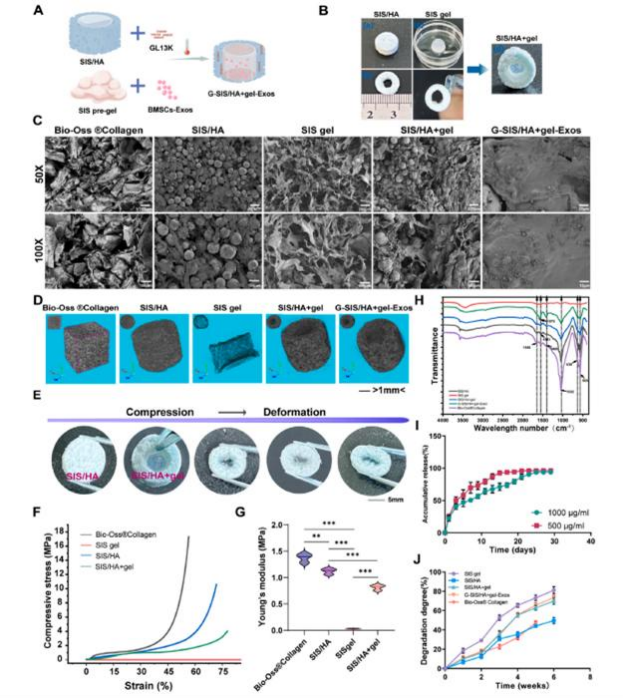
Fabrication and characterization of the coaxial G-SIS/HA+gel-Exos scaffold. (A) Schematic of scaffold synthesis. (B) Morphological structure and assembly. (C) SEM images of different groups. (D) Micro-CT images showing scaffold porosity and internal architecture. (E) Compression and deformation test. (F-G) Mechanical strength data. (H) FTIR spectra. (I) GL13K release profile. (J) Degradation rate over time. Adapted from Ma et al. (2024), *Bioactive Materials*, under CC BY-NC-ND 4.0. ^26^

**Figure 8:**
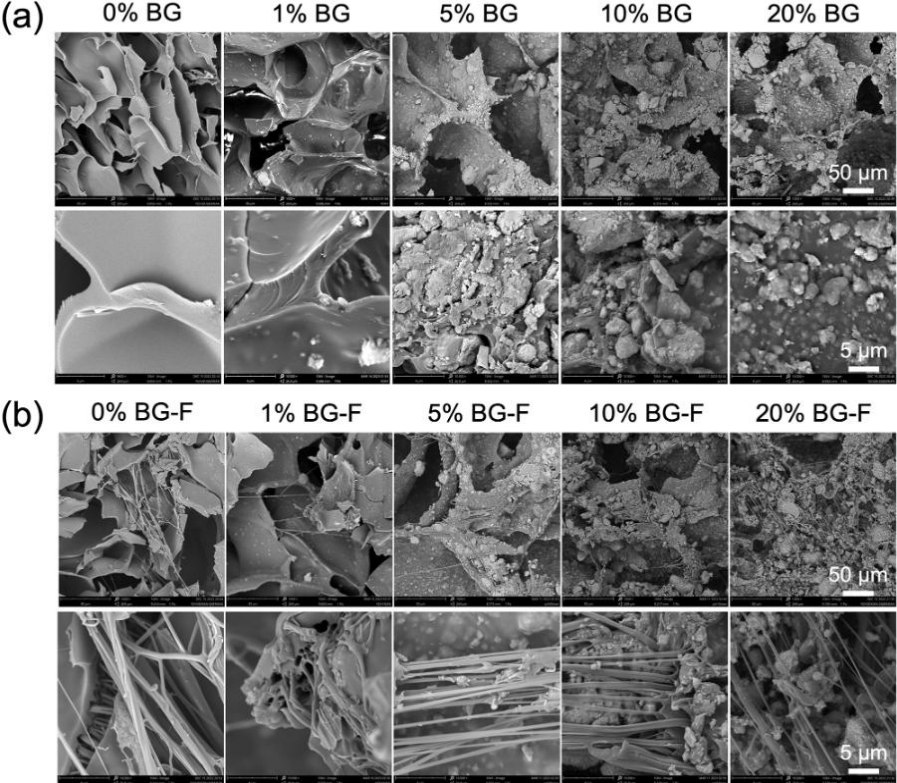
SEM images of GelMA-based hydrogels with varying concentrations of bioactive glass (BG) and coaxial PCL@GelMA fibers (BG-F). (a) SEM of 0-20% BG showing surface roughening with increased BG content. (b) SEM of BG-F scaffolds with enhanced fiber alignment and BG particle distribution. Adapted from Yu et al., 2024 (*J. Nanobiotechnol.*), under CC BY 4.0.^70^

### Preclinical and clinical performance of composite scaffolds

The translation of composite bioactive scaffolds from bench to bedside relies heavily on their proven efficacy in preclinical and clinical studies. These studies evaluate not only the scaffold’s osteoconductive and osteoinductive capacity, but also its ability to integrate within native tissue, support vascularization, and demonstrate predictable resorption profiles.

### Preclinical Studies in Mandibular and Alveolar Models

Preclinical testing in animal models provides critical insight into scaffold performance under physiological loading conditions and bone remodeling dynamics, serving as essential validation steps before clinical translation. For example, Kim et al. (2024) demonstrated that 3D-printed polydiolcitrate-HA scaffolds implanted in rabbit mandibular defects significantly enhanced bone volume and vascular infiltration within 8 weeks ^14^. Similarly, Yao et al. (2024) reported that collagen/gelatin/nano-β-TCP scaffolds promoted new bone formation and accelerated osteoid mineralization in a rat alveolar bone defect model ^13^. These findings highlight the importance of scaffold composition in promoting both structural and biological integration in load-bearing oral environments.

Injectable scaffolds have also demonstrated promising results in preclinical evaluations, offering advantages in terms of minimally invasive delivery and conformability to irregular defect geometries. In a calvarial defect model, He et al. (2025) observed that their chitosan-puerarin-silica hydrogel scaffold not only improved osteogenic differentiation but also reduced macrophage-mediated inflammation, indicating important immunomodulatory benefits that are essential for optimal healing in oral and maxillofacial surgery contexts ^15^. Comparative studies have provided valuable insights into the efficacy of tissue-engineered approaches versus traditional bone grafting methods. Shahnaseri et al. (2020) compared autogenous bone grafts with tissue-engineered constructs comprising HA/β-TCP scaffolds loaded with adipose-derived stem cells in a canine model of alveolar cleft ^128^. Radiographic densitometry and histologic evaluation up to 90 days post-grafting demonstrated comparable bone density outcomes between the two approaches, highlighting the potential clinical utility of stem cell-based composite grafts for alveolar reconstruction and suggesting that engineered scaffolds may serve as viable alternatives to autografts.

**Box 4:**
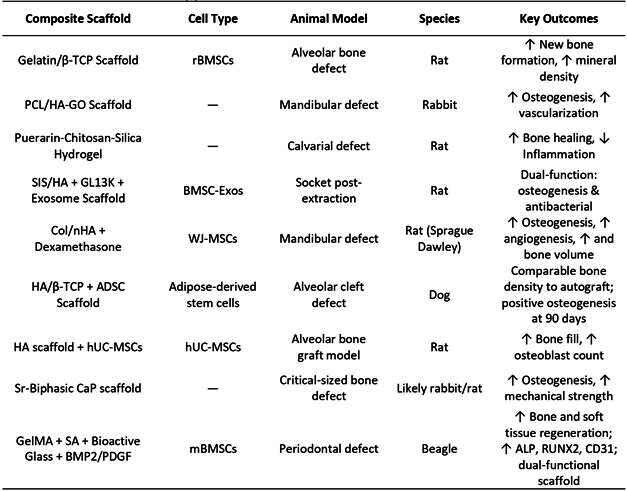
Preclinical scaffold summary ^13^ ^-^ ^15^ ^,^ ^26^ ^,^ ^106^ ^,^ ^128^ ^,^ ^130^ ^,^ ^131^ ^,^ ^132^

The role of growth factors in scaffold-mediated bone regeneration has been extensively investigated in preclinical models. Uribe et al. (2022) conducted a comparative study using a rat calvarial critical-sized defect model to evaluate the osteogenic performance of rhBMP-2 combined with different scaffold carriers, including β-TCP, hydroxyapatite (HA), bovine xenograft, and collagen sponges ^129^. Their findings revealed that rhBMP-2 enhanced new bone formation when used with all carriers but notably showed favorable healing scores with β-TCP and autograft combinations at 4 and 8 weeks. However, by 8 weeks, the differences in bone formation among materials were not statistically significant, suggesting that scaffold composition may influence early-stage outcomes more than long-term bone maturity. Additional preclinical evidence supports the efficacy of polymer-ceramic composite systems in oral bone regeneration. Recent studies have demonstrated that PLGA-bioactive glass composite scaffolds show promising results in rat models of alveolar bone defects, with PLGA scaffolds containing 10% bioactive glass seeded with stem cells from human exfoliated deciduous teeth promoting substantial new bone formation and osteocalcin expression six months post-implantation. These findings support the relevance of scaffold selection in early regenerative stages, particularly in oral and maxillofacial surgery contexts where timing of bone maturation is crucial for subsequent implant placement or secondary surgical procedures.

### Clinical Applications of Composite Scaffolds

Although most composite scaffolds remain at the preclinical stage, a select few have progressed to human application, particularly in dental implantology and ridge augmentation procedures ^133^ ^,^ ^134^ ^,^ ^135^. Bioactive glass-based scaffolds combined with autogenous grafts have been used successfully in sinus lift procedures, demonstrating improved bone height and enhanced implant stability in clinical settings. PCL/HA composites have also been applied in guided bone regeneration (GBR) protocols, where they effectively maintain space and support new bone fill in mandibular ridge preservation cases. These early clinical applications provide valuable insights into the practical implementation of composite scaffold technology in oral and maxillofacial surgery. However, widespread clinical adoption remains limited due to several significant challenges, including regulatory hurdles, cost considerations, and the critical need for comprehensive long-term safety data. Few composite scaffolds have received FDA approval specifically for craniofacial use, highlighting a substantial gap between laboratory potential and real-world clinical implementation. This regulatory landscape underscores the complexity of translating advanced biomaterial research into routine clinical practice. Recent clinical studies have begun to address this translation gap with promising results. Jeong et al. (2022) conducted a prospective clinical study involving eight patients with complex zygomatico-maxillary defects who underwent reconstruction using 3D-printed patient-specific PCL/β-TCP scaffolds ^136^.

The scaffolds demonstrated satisfactory volume conformity with an average of 79.71% and new bone formation with a mean bone volume fraction of 23.34% after 6 months, as evaluated by CT-based volumetric and radiodensity analyses. The authors concluded that this scaffold type offers a biocompatible, moldable, and structurally supportive alternative for maxillofacial bone reconstruction, particularly in anatomically complex regions where traditional grafting approaches may be limited. Furthermore, Ivanovski et al. (2024) conducted a detailed case report using a patient-specific 3D-printed polycaprolactone (PCL) scaffold for staged alveolar bone augmentation prior to dental implant placement ^137^. The scaffold demonstrated excellent fit, biocompatibility, and structural integration, leading to significant volumetric bone gain and stable implant placement. This case illustrates the substantial translational potential of composite scaffolds in scaffold-guided bone regeneration (SGBR) protocols for maxillofacial applications, suggesting that personalized scaffold approaches may become increasingly viable for complex reconstructive cases. These clinical experiences provide crucial evidence for the feasibility and efficacy of composite scaffolds in real-world maxillofacial surgery applications**.**

Some patient-specific implants reported in maxillofacial reconstruction are placed under custom-made medical device exemptions, which are intended for an individual patient and not equivalent to market-approved products, where evidence requirements and post-market surveillance differ from approved devices (e.g., FDA 510(k)/PMA; CE; ANVISA approvals). We clearly indicate when studies used custom-made devices versus commercially approved materials so readers can interpret safety/efficacy claims appropriately.

### Limitations and Considerations in Translational Use

Despite the promising results demonstrated in preclinical studies, significant challenges remain in translating scaffold technologies into routine clinical protocols for maxillofacial bone regeneration. One of the primary concerns involves the considerable variability in scaffold degradation rates across different patients, which can be influenced by individual metabolic factors, immune responses, and local tissue conditions ^138^ ^,^ ^139^ ^,^ ^140^. This variability makes it difficult to predict optimal healing timelines and may require personalized treatment protocols that are currently challenging to implement in clinical practice. Manufacturing and quality control present additional substantial hurdles, particularly in scaling up production while maintaining consistent quality, sterility, and shelf-life without compromising the bioactivity of incorporated growth factors or bioactive molecules ^141^.

The complex nature of composite scaffolds, which often contain multiple components with different storage requirements and stability profiles, further complicates the manufacturing process and regulatory approval pathways. These technical challenges contribute to increased costs and extended development timelines that can hinder widespread clinical adoption. Furthermore, the translation from animal models to human applications introduces additional complexity, as scaffold performance may differ significantly between species due to fundamental differences in immune responses, bone turnover rates, and defect geometry characteristics. The human healing response, particularly in the oral and maxillofacial region, involves unique factors such as exposure to oral bacteria, saliva, and complex masticatory forces that are difficult to replicate accurately in animal models. For mandibular applications specifically, the dynamic occlusal loading and varying masticatory force patterns further complicate scaffold evaluation, necessitating the development of more sophisticated load-bearing preclinical models that can better predict human clinical outcomes. These considerations underscore the need for carefully designed clinical trials and long-term follow-up studies to establish the safety and efficacy of composite scaffolds in real-world maxillofacial surgery applications.

**Box 5:**
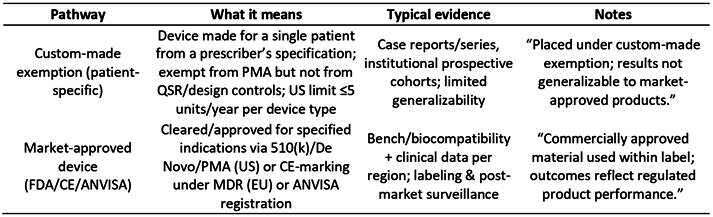
Regulatory status shorthand used in this review - custom-made exemptions versus market-approved devices (FDA/EU/ANVISA) ^146^

### Summary of Translational Insights

Preclinical data support composite scaffolds that jointly address mechanics, angiogenesis, and immune tone in the jaw. For translation, priorities include GMP-compatible fabrication with tight QA/QC (sterility, residuals, batch reproducibility), validated clinical endpoints (time-to-implant, volumetric bone gain, functional load tolerance), and patient-centered outcomes (pain, re-entry morbidity, quality of life). Cost-effectiveness versus autograft/allograft pathways and clear regulatory strategies (device vs combination product) will determine real-world adoption.

Current preclinical data comprehensively support the feasibility of composite scaffolds for alveolar and mandibular bone repair, particularly in customized, dual-functional designs that address both mechanical and biological requirements. The evidence from animal studies demonstrates that these advanced biomaterial systems can effectively promote bone regeneration while providing necessary structural support during the healing process. Early clinical experiences, though limited in scope, are encouraging and provide valuable proof-of-concept data for the potential of composite scaffolds in oral and maxillofacial surgery applications. However, the transition from promising preclinical results to widespread clinical implementation requires substantial additional validation through larger, multi-center trials and comprehensive long-term evaluation studies. These studies must address not only efficacy outcomes but also safety profiles, patient-specific variability, and long-term stability of regenerated bone tissue. Future success in clinical translation will fundamentally depend on developing GMP-compliant fabrication processes that ensure consistent quality and reproducibility at scale, generating robust biocompatibility data that satisfy regulatory requirements, and creating scaffold designs specifically tailored to the unique biomechanical demands of oral and maxillofacial surgery.

The integration of advanced manufacturing technologies, personalized medicine approaches, and sophisticated biomaterial design principles positions composite scaffolds as a promising solution for complex maxillofacial reconstruction challenges. As the field continues to evolve, the convergence of materials science, tissue engineering, and clinical expertise will be essential for realizing the full therapeutic potential of these innovative regenerative technologies in improving patient outcomes and quality of life.

### Future directions and challenges

### Customization and Patient-Specific Design

As mandibular and alveolar bone defects demonstrate significant variation in size, shape, and biomechanical demands across individual patients, there is an increasingly critical need for patient-specific scaffold designs that can address these unique anatomical and functional requirements. The heterogeneous nature of maxillofacial defects, ranging from minor alveolar ridge deficiencies to complex segmental mandibular reconstruction cases, necessitates a personalized approach that moves beyond the traditional one-size-fits-all paradigm toward precision medicine applications in oral and maxillofacial surgery.

Recent advances in 3D printing technologies, computer-aided design and manufacturing (CAD/CAM) systems, and sophisticated intraoperative imaging modalities offer unprecedented opportunities to fabricate scaffolds that conform precisely to individual defect geometry while optimizing mechanical properties for specific loading conditions ^142^. These technological innovations enable the creation of patient-specific scaffolds that can replicate complex anatomical contours, accommodate irregular defect shapes, and provide tailored porosity gradients that match local tissue requirements ^142^. The integration of advanced imaging techniques such as cone-beam computed tomography (CBCT) and magnetic resonance imaging (MRI) with computational modeling allows for the prediction of stress distribution patterns and optimization of scaffold architecture before surgical intervention ^142^. However, translating this sophisticated customization capability into routine clinical practice requires the seamless integration of patient imaging data with biocompatible and printable biomaterials while ensuring consistent mechanical robustness and maintaining sterility throughout the manufacturing process. This integration presents significant challenges in terms of workflow optimization, quality control, regulatory compliance, and cost-effectiveness ^143^. The development of standardized protocols for data acquisition, processing, and manufacturing will be essential for the widespread adoption of patient-specific scaffold technologies in oral and maxillofacial surgery, ultimately enabling more predictable outcomes and improved patient satisfaction through truly personalized regenerative treatments ^143^.

CBCT-to-CAD pipelines coupled with finite element modeling (FEM) can pre-screen lattices against mandibular load cases (clench, unilateral mastication), minimizing stress shielding and tuning strut gradients for 100-200 MPa cortical-analog support. Emerging AI-assisted topology optimization can co-optimize pore architecture for perfusion (Darcy flow targets) and mechanics, accelerating iteration toward patient-specific designs. ^143^

### Integration of Biofunctionality and Drug Delivery

The next generation of composite scaffolds for maxillofacial bone regeneration must transcend traditional structural support roles to incorporate sophisticated biofunctional capabilities that address the complex clinical challenges encountered in oral and maxillofacial surgery. Future scaffolds must not only support osteogenesis but also seamlessly integrate multiple functionalities, such as antibacterial, anti-inflammatory, or anticancer drug delivery systems that can respond to specific clinical scenarios. This multifunctional approach is particularly relevant for post-tumor resection cases where the risk of local recurrence and opportunistic infections remains significantly elevated, requiring simultaneous therapeutic intervention alongside bone regeneration ^145^.

The development of dual-delivery and multi-drug release systems using sophisticated nanocarriers embedded within the scaffold matrix represents a promising avenue for addressing these complex clinical needs. These advanced delivery platforms can incorporate different therapeutic agents with distinct release profiles, allowing for sequential or simultaneous delivery of antibiotics during the early healing phase, anti-inflammatory agents during the intermediate inflammatory response, and growth factors to promote sustained osteogenesis. The integration of smart nanocarriers, such as pH-responsive microspheres or temperature-sensitive liposomes, enables targeted drug release in response to local tissue conditions or pathological changes. However, the clinical translation of these sophisticated dual-delivery systems requires precise control over release kinetics to ensure therapeutic efficacy while minimizing systemic toxicity, comprehensive biocompatibility testing of all incorporated components, and extensive regulatory approval processes that address the complexity of combination products. The challenge lies in achieving optimal drug loading capacities while maintaining scaffold mechanical properties, ensuring stable drug activity throughout the storage and implantation period, and developing reliable methods for monitoring drug release in vivo. Successfully addressing these technical and regulatory challenges will enable the development of brilliant scaffold systems that can adapt to changing clinical conditions and provide comprehensive therapeutic solutions for complex maxillofacial reconstruction cases ^144^ ^,^ ^145^.

### Long-Term Safety and Degradation Control

Controlling scaffold degradation to match bone regeneration rates precisely remains one of the most critical challenges in the clinical translation of composite scaffolds for maxillofacial applications. The temporal coordination between scaffold resorption and new bone formation is essential for successful regenerative outcomes, as both premature degradation and prolonged persistence can lead to suboptimal healing responses or adverse inflammatory reactions. Premature scaffold degradation can result in mechanical instability and void formation before adequate bone matrix has been deposited, while excessively slow resorption may impede natural bone remodeling processes and potentially trigger chronic foreign body responses that compromise long-term integration ^145^.

The complexity of this temporal matching is further amplified in the oral and maxillofacial region, where scaffolds must withstand dynamic mechanical loading while maintaining biological functionality throughout the extended healing period. The unique biomechanical environment of the jaw, characterized by repetitive masticatory forces and complex stress distributions, requires scaffolds with carefully engineered degradation profiles that can provide sustained mechanical support during the critical early healing phases while gradually transferring load to newly formed bone tissue as regeneration progresses ^145^.

There is an urgent need for comprehensive in vivo studies evaluating long-term outcomes over extended follow-up periods, including detailed assessment of immune response patterns, scaffold-bone integration quality, and resorption kinetics, specifically in load-bearing oral and maxillofacial surgery sites. These studies must address critical questions regarding the long-term biocompatibility of degradation products, the potential for delayed inflammatory responses, and the quality of the bone-scaffold interface over time. Additionally, standardized protocols for monitoring scaffold degradation and bone regeneration in clinical settings are essential for optimizing treatment protocols and ensuring patient safety. The development of predictive models that can forecast degradation behavior based on patient-specific factors such as bone turnover rates, immune status, and mechanical loading patterns will be crucial for advancing personalized regenerative medicine approaches in maxillofacial reconstruction ^145^.

### Regulatory and Manufacturing Barriers

From a translational perspective, one of the most significant obstacles preventing widespread clinical adoption of composite scaffolds remains the complex regulatory approval process and the challenge of establishing scalable manufacturing protocols under Good Manufacturing Practices (GMP) standards. The regulatory landscape for composite scaffolds is particularly complex because these products often fall into multiple regulatory categories, potentially being classified as combination products that include biomaterials, drugs, and sometimes biological components, each with distinct regulatory requirements and approval pathways ^146^.

Scaffold systems must demonstrate rigorous batch consistency, reproducibility, and sterility across all manufacturing steps, along with comprehensive preclinical and clinical data that address safety, efficacy, and quality control parameters. The challenge is compounded by the need to maintain the bioactivity of incorporated therapeutic agents while ensuring consistent mechanical properties and degradation characteristics across different production batches. This requires sophisticated quality control systems, validated analytical methods, and robust manufacturing processes that can accommodate the complex multi-component nature of composite scaffolds while meeting stringent regulatory standards ^146^.

The transition from laboratory-scale synthesis to commercial-scale production presents additional technical challenges, including the scaling of 3D printing processes, maintaining sterility in complex manufacturing workflows, and ensuring cost-effectiveness while preserving product quality. Many promising scaffold technologies remain trapped in the "valley of death" between research and clinical application due to these manufacturing and regulatory hurdles ^146^.

Effective collaboration among researchers, clinicians, regulatory bodies, and manufacturing specialists is essential to streamline approval pathways and develop standardized guidelines for composite scaffold evaluation. The establishment of regulatory science initiatives, the development of consensus standards for scaffold characterization, and the creation of expedited approval pathways for breakthrough technologies will be crucial for accelerating the translation of these promising regenerative therapies from bench to bedside, ultimately benefiting patients who require complex maxillofacial reconstruction ^146^.

### Sustainability and Smart Biomaterials

Emerging trends in composite scaffold development are increasingly focused on sustainability and the incorporation of intelligent biomaterial systems that represent the next frontier in regenerative medicine for maxillofacial applications. The growing emphasis on environmental responsibility has led to increased interest in sustainable, naturally-derived polymers that can reduce the ecological footprint of medical devices while maintaining superior biological performance. These bio-based materials, sourced from renewable resources such as marine organisms, plant extracts, and agricultural waste products, offer the dual advantages of reduced environmental impact and enhanced biocompatibility due to their natural origin and similarity to native biological structures. Simultaneously, the incorporation of stimuli-responsive or innovative biomaterials that can dynamically adapt to the biological environment represents a paradigm shift toward brilliant scaffold systems. These sophisticated innovative systems can respond to various biological stimuli, including pH changes, temperature fluctuations, or specific enzymatic activity patterns that occur during different phases of bone healing. This responsiveness enables on-demand release of therapeutic agents precisely when and where they are needed, providing dynamic remodeling support that adapts to the changing requirements of the healing tissue environment ^147^.

The implementation of innovative biomaterial systems is desirable for complex oral and maxillofacial surgery cases, where the healing environment is highly dynamic and variable. For instance, pH-responsive scaffolds can release antibiotics in response to bacterial infection-induced acidosis, while enzyme-responsive systems can deliver growth factors in response to specific proteolytic activities associated with tissue remodeling. Temperature-responsive materials can provide controlled drug release triggered by inflammatory responses, creating truly adaptive therapeutic platforms. These innovations toward sustainable and intelligent scaffold design represent a convergence of environmental stewardship, advanced materials science, and personalized medicine that promises to revolutionize maxillofacial reconstruction. The successful integration of sustainability principles with smart material functionality will be essential for developing next-generation scaffold systems that are not only clinically effective but also environmentally responsible and economically viable for widespread clinical implementation148.

## Conclusion

Bioactive composite scaffolds are emerging as credible alternatives to conventional grafts for mandibular and alveolar reconstruction because they pair mechanical competence with biological instruction. This review synthesized jaw-specific biology (angiogenesis and osteo-immunomodulation), mapped how composition controls properties and outcomes across major classes (e.g., collagen/β-TCP, PCL/HA, GelMA/BG, PLGA/β-TCP/Mg, Sr-BCP, CaCO₃ hybrids, and patient-specific PCL/β-TCP), and compared fabrication routes (extrusion, DLP, MEW, porogen-leaching) together with their technology-readiness levels (TRLs). We also clarified when devices fall under custom-made exemptions versus market-approved pathways, critical context for interpreting generalizability. To translate promise into routine OMFS care, three priorities must align: manufacturability and quality (GMP-grade materials, validated sterilization, tight batch reproducibility, and scalable production), evidence-standardized mandibular/maxillary models and quantitative readouts that couple revascularization with bone formation, followed by human studies that extend beyond custom-made use, and digital integration (CBCT-to-CAD/CAM with FEM/AI-assisted design to tune stiffness, hierarchical porosity, and drug/ion delivery for site-specific biomechanics). Achieving these will reduce procedures, shorten rehabilitation, and better restore mastication, speech, and facial aesthetics. Biologically informed, digitally planned, and regulatorily robust composite scaffolds are the most direct route from innovation to predictable patient benefit.

## Data Availability

The research data are not available
